# DNA Methylation, Epigenetics, and Evolution in Vertebrates: Facts and Challenges

**DOI:** 10.1155/2014/475981

**Published:** 2014-01-16

**Authors:** Annalisa Varriale

**Affiliations:** Laboratory of Molecular Evolution, Stazione Zoologica Anton Dohrn, 80121 Naples, Italy

## Abstract

DNA methylation is a key epigenetic modification in the vertebrate genomes known to be involved in biological processes such as regulation of gene expression, DNA structure and control of transposable elements. Despite increasing knowledge about DNA methylation, we still lack a complete understanding of its specific functions and correlation with environment and gene expression in diverse organisms. To understand how global DNA methylation levels changed under environmental influence during vertebrate evolution, we analyzed its distribution pattern along the whole genome in mammals, reptiles and fishes showing that it is correlated with temperature, independently on phylogenetic inheritance. Other studies in mammals and plants have evidenced that environmental stimuli can promote epigenetic changes that, in turn, might generate localized changes in DNA sequence resulting in phenotypic effects. All these observations suggest that environment can affect the epigenome of vertebrates by generating hugely different methylation patterns that could, possibly, reflect in phenotypic differences. We are at the first steps towards the understanding of mechanisms that underlie the role of environment in molding the entire genome over evolutionary times. The next challenge will be to map similarities and differences of DNA methylation in vertebrates and to associate them with environmental adaptation and evolution.

## 1. Environmental Epigenetics and DNA Methylation

In vertebrates, cytosine DNA methylation is a heritable epigenetic modification that occurs mostly at the CpG dinucleotides except for the CpGs in CpG islands [1]. Recently, it has become extremely attractive given its involvement in a diverse range of cellular functions including tissue-specific gene expression, cell differentiation [2], development [3, 4] and reprogramming ([5] see references therein), genomic imprinting, X chromosome inactivation, and regulation of chromatin structure and disease states [6–9]. Notably, the epigenome contains hypervariable regions that could be a source of cellular diversity [10] or could underlie disease states or provide an engine for neutral selection at cell or tissue level [11]. Such hypervariability might be influenced by metabolite fluctuations, temperature variation, and other environmental agents that exert their action on chromatin-modifying enzymes and gene regulation [12–15]. A clear example of how environment plays an important role in shaping the epigenome is represented by monozygotic twins, who are epigenetically indistinguishable early in life but with age exhibit substantial differences in epigenetic markers [16]. The effect of environment on epigenome changes is evident even in flowering plants, where vernalization requires methylation of specific histone arginine and lysine residues [17, 18], revealing a link between temperature and chromatin state.

These are examples of how environmental cues of short duration can cause small epigenetic modifications having a direct effect on genes and therefore visible on phenotype. Different is the case of genomes exposed to certain stimuli for evolutionary times (see below).

### 1.1. Environmental Epigenetics Associated with Diseases

The epigenetic state is easily affected by environmental factors as exposure to xenobiotics, social behavior, metabolism, and nutritional deficiencies that may exert their effects later in life, during critical periods of development [19], or may be transmitted transgenerationally to the offspring [20]. Due to the difficulty of establishing the right contribution of genetic and environmental elements and since the elimination of the environmental factor could determine the reversion of epigenetic modifications, the role of environmental factors in epigenetic changes is still matter of debate [21]. However, at present, various human diseases such as neurologic disorders (e.g., Rett syndrome and (alpha)-thalassemia X-linked mental retardation; see [22]), pathologies associated with loss of imprinting (e.g., Prader-Willi, Angelman, and Beckwith-Weidemann syndromes), congenital malformation, and aging are considered consequences of epigenetic alterations [23]. In addition, changes in genomic DNA methylation and histone acetylation patterns [24, 25] are connected with neoplasia [26]. In this context, it is possible to hypothesize that in the next future the epigenome could become a therapeutic target and that personalized medicine will probably be affected by epigenetic differences between individuals.

### 1.2. Environmental Epigenetics Associated to Ecology

Recently, it has been highlighted how continue exposition to environmental stress can represent a major force behind the evolutionary creation of new species through effects on epigenome [27]. The authors draw the general conclusions that under new conditions, epigenome adapts and can increase the rate of adaptive evolution, by activating silent genes and through heritable variants. Modifications in the environment can induce epigenetic modifications and, in turn, transcription state changes that are a source of phenotypic variability that may increase the “adaptative potential” [28]. In addition to transcriptional changes, transposon activity is another source of genetic diversity. Two different authors [29, 30] speculate that epigenetic instability in response to environmental changes can promote transposable elements activity whose outcomes may include sexual isolation and speciation. The renewal of gene networks, in fact, allows the arousal of new species establishing a link between environmental changes, natural selection, and evolution. These conclusion, represent relevant insights for further studies aimed at clarifying how epigenetic regulation affects natural population variations [30–32].

## 2. Environmental Epigenetics and DNA Methylation in the Context of Vertebrate Genomes

Cytosine DNA methylation is widespread in most major eukaryotic groups, including plants, animals, and fungi. However, despite its wide distribution, neither the biological function of gene body methylation nor the mechanisms by which genes are recognized by DNA methylation systems are fully understood yet. Although its role in gene silencing appears to be conserved, the levels and genomic patterns of DNA methylation show considerable variation among taxa [10, 33–39]. For example, vertebrate genomes are extensively methylated [10, 35, 36]. In contrast, invertebrate genomes display variable levels of DNA methylation [33, 40–43]. Experimental discoveries demonstrating high variability in the patterns of DNA methylation in different taxa are an interesting starting point for studying divergence in methylation pathways and regulatory mechanisms and raise questions such as what is the meaning of different amounts of DNA methylation among animal species? What could be its functions and evolution in different animal genomes? However, determining precise genome scale methylation patterns has been a challenge for complex genomes until the recent development of high-throughput sequencing technology, which has allowed the analysis of the methylomes of single organisms, such as human [1], chicken [44], or arabidopsis [38–45].

Recently, two surveys focused on methylation patterns in a number of eukaryotes [37, 46], both reporting that methylation in genes, exons, transposons, and repetitive DNA shows peculiar patterns among the tested organisms. DNA methylation, in fact, is unevenly distributed in the genome, since the heterochromatin region, transposons, and repetitive sequences are usually hypermethylated, whereas the flanking regions of genes are methylated at a relatively low level compared with the gene body regions [47, 48].

### 2.1. DNA Methylation Levels in Vertebrate Genomes Are Correlated with Body Temperature

**Figure 1:**
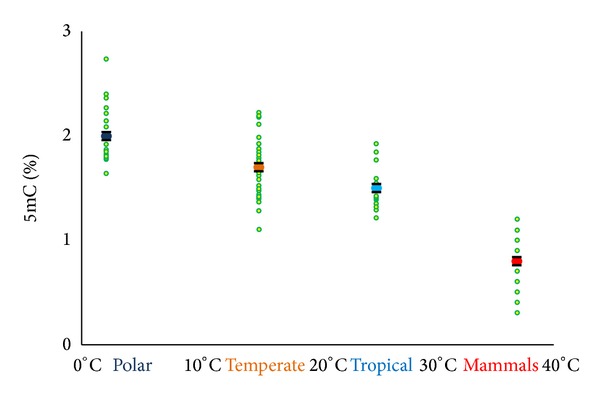
Correlation between 5mC level and body temperature. The green points represent species, whereas the middle points are the averages of polar, temperate, tropical fishes, and mammals. Standard error bars are shown. Modified from [35].

In our laboratory, we were interested in understanding the meaning and evolution of different patterns and levels in the living species and their meaning in an evolutionary context and the role of environment in epigenetic variation and phenotypic evolution. So we began to analyze the global methylation levels in the DNA of a number of vertebrate species belonging to both cold-blooded and warm-blooded vertebrates in order to clarify the correlation existing between DNA methylation and body temperature [35, 36, 49]. At that time, genome wide methods were not available yet, so we chose for our analyses the reversed phase high performance liquid chromatography that allowed us to explore the global DNA methylation level in a very large number of species in a relatively short time. The results obtained on both cold- and warm-blooded vertebrates are summarized in Figure 1. Since fishes live in a thermostable environment and mammals have body temperatures that are stable and well established, we grouped species into four categories depending on their living temperature. The first group ranges from 0°C to 10°C and includes polar and subantarctic species and the two middle groups range from 10°C to 20°C and from 20°C to 30°C and contain temperate and tropical fishes, respectively, whereas the mammalian group goes from 30°C to 40°C. Average methylation level and standard error for each group are reported, showing the inverse relationship between 5mC and living temperature of the groups.

**Figure 2:**
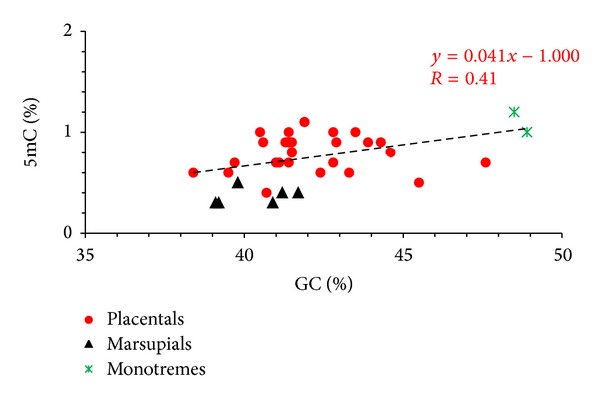
Plot of 5mC levels against GC levels in mammalian genomes. We have graphically distinguished placentals (red dots), marsupials (black triangles), and monotremes (green crosses). Species and experimental values of GC and 5mC are listed in Table 1; values are from [49] and Varriale and Bernardi, unpublished data.

**Figure 3:**
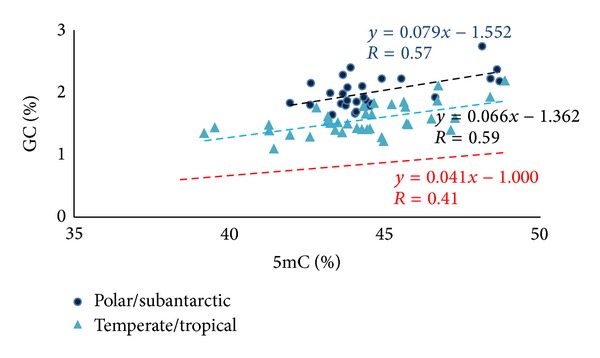
Plot of 5mC levels against GC levels for the genomes of polar fishes (dark blue circles) and temperate/tropical fishes (light blue triangles). The regression line (broken red line) for the mammalian DNA (data from [49] and unpublished data; see Figure 2) was reported as a reference. Modified from [35].

The observed variability in DNA methylation levels within groups could much probably be due to differences in genomic GC level (as shown in Figures 2 and 3) to which 5mC is correlated and partly to other intrinsic genomic differences, such as presence of repetitive and satellite sequences and genome size (*c*-value) [40]. A positive, linear correlation holds between the frequency of CpG and 5mC and the GC levels. Indeed, methylation linearly increases with genome GC in the case of all vertebrates tested, but 5mC levels of warm-blooded vertebrates were systematically lower than those of cold-blooded vertebrates (even when considering two species, mammal and fish, with same GC level).

### 2.2. Warm-Blooded and Cold-Blooded Vertebrates

Mammals are known to be homeotherms for their ability to maintain a high and constant body temperature through homeostasis, so that they were traditionally called also warm-blooded vertebrates. The class includes three different superorders with high but slightly different body temperatures, marsupials and monotremes centered around 33°–35°C and placentals with 37°-38°C. Since during evolution high body temperatures have favored loss of CpG and of methylation, the comparison of mammalian DNA methylation levels (5-mC) versus cold-blooded vertebrates was required to shed light about the influence of temperature on epigenomes over evolutionary times. The first analyses were carried out in 1997: the authors [49] analyzed the level of 5mC in DNA in placental mammals together with monotremes and compared them with birds and cold-blooded vertebrates. They demonstrated that (i) monotremes are more GC rich and 5mC rich in comparison to other mammals; (ii) mammals and birds have a comparable 5-mC level; (iii) 5-mC levels of mammals and birds are lower compared to fishes and amphibians. The cause of the two independent, convergent, and compositional transitions loading to the genomes of mammals and birds can be visualized as the permanent high body temperature. Many years later, we extended these analyses by adding the first time data for DNA methylation in marsupials and some more placentals (unpublished data; see Table 1 and Figure 2). Interestingly, marsupials, in spite of having a body temperature similar to that of monotremes, have the lowest 5mC amount among all the vertebrates explored until now. With much probability, this is due to their extremely low genomic GC-level, similar to that of rodents, and also having a low methylation level. In fact, GC and 5mC levels are positively correlated [50].

So, we can observe that, from one side, body temperature is a very strong factor influencing whole genomic methylation of vertebrates, and differences due to it are evident in ranges of 10–15 degrees (such as in the comparisons of tropical/polar fishes mammals/fishes). From the other side, when we compare species with smaller differences of temperature (such as in the comparison of placental/marsupials or monotremes, or among polar fishes), we have to take into account other factors, such as GC heterogeneity (see also Section 2.5). Along this line, it is possible to speculate that methylation can be modified during evolutionary times by environment, but it is reasonable that modifications, in both genic and nongenic regions, cannot go beyond a certain level, given the important functions methylation. This could be the explanation of the fact that when we consider mammals of 30°C or 37°C we do not observe huge differences in DNA methylation and other factors must be taken into account. In the same way, if we analyze different tissues or individuals belonging to the same species, differences can be even smaller and can be due to differences of gene expression or GC3 [51] and so undetectable over the whole genome by analytical techniques.

### 2.3. Polar and Temperate/Tropical Fishes

Teleost fishes are extremely attractive for the study of evolution since they couple an extremely huge number of species with a high level of biodiversity. The main advantage for investigating the organism-environment interface is that wild species are cold-blooded thermoconformers, meaning that their body temperature reflects the external temperature. We considered this feature much relevant to our studies because it is pretty easy to find an enormous number of species adapted to living in extremely different habitats. We analyzed fishes living in different habitats with different temperatures obtaining, as result, a negative correlation between methylation and temperature when comparing the 5mC levels in fishes living at different environments, independently of phylogenetic distance. More in particular, we compared polar fishes with temperate and tropical fishes having more than 20°C different body temperatures. Interestingly, both Antarctic and Arctic fishes, belonging to different taxonomic orders, but having in common an extremely cold habitat, exhibit the highest values of DNA methylation among all fishes studied. In Figure 3 there are displayed the results of DNA methylation analysis in polar and temperate/tropical fishes. In addition, when we restricted our comparison to temperate and tropical fishes, we found again a little but significant difference with the same trend (see Figure 1). These results were consistent and reinforced the hypothesis of environmental selection of the genome, since the variation in epigenome is clearly correlated with variation in genome. Along this line, it must be reminded that environment has also a direct influence on genome composition [52].

### 2.4. Influence of the Environment on Fish Transcriptome

Work in fish has so far evidenced two kinds of environmental response: short-term phenotypic responses and evolutionary responses. For the first case, studies carried out in the common carp, *Fundulus*, and in the catfish have demonstrated that a surprisingly large fraction of the genome is involved in the phenotypic transition to the cold-adapted state and several important new candidate genes were identified for physiological assessment [53, 54]. Results indicated that some patterns of gene expression exceed levels of variation that are expected under the neutral hypothesis and seem to be an adaptation to the environment. For the second case, the ability to make comparisons between fish species that can survive in cooling temperatures and their sister taxa that die at temperatures below 20°C was a key strength of fish environmental genomics. Chen et al. [55] analyzed the transcriptome of a stenothermal Antarctic fish, *Dissostichus mawsoni*. Their analysis demonstrates that cold exposure of a poikilotherm that naturally experiences environmental cooling involves the regulation of a very large number of genes. In another very recent study [56], authors compared orthologous sequences of two closely related zoarcid fishes inhabiting different latitudinal zones (Antarctica and temperate sea) and detected significant differences in codon usage, the cold species having lower GC3 in the wobble position.

These evidences, coupled with the observation that DNA methylation is involved in the control of gene expression, give the possibility of speculating that an evolutionary connection between environment, gene expression, and adaptation is possible.

Identifying exactly which genes control susceptibility to environmental stress remains challenging. The underlying determinants of thermal plasticity are also interesting in the context of stenothermal species, which may either lack certain classes of genes or are unable to regulate their expression in response to changing temperature. In the case of Antarctic fishes, cold has a pervasive effect on the transcriptome giving rise to a complex adaptive phenotype that not only improves physiological performance in the cold but also promotes thermotolerance in this extremely harsh environment.

### 2.5. Implications of Different DNA Methylation Levels in Vertebrates

Maintaining methylation is a dynamic process that involves on one side the environment and on the other side the enzymes and factors whose function is to keep methylation to a certain amount. Methylation levels in fact could reach a peculiar level corresponding to the amount required by an organism to maintain safe its biological functions. This last hypothesis is stressed by three facts: (i) the ancestors of vertebrates, the cephalochordates and urochordates, are characterized by genomes that are neither GC-rich nor strongly methylated; (ii) mammals belonging to orders separated from each other by 100 million years display very similar methylation levels. Because of the star-like phylogeny of mammalian orders, this situation indicates that the ancestral mammalian genome also showed the same levels of extant mammals. This conclusion contradicts also the hypothesis that the vertebrate genome originally was strongly methylated (and GC-rich) and subsequently underwent a monotonous decay that, otherwise, should have completely erased methylation and CpG nucleotides from the genomes [57]; (iii) it is known that organisms require the right amount of methylation and aberrant levels cause serious damages due to changes in gene expression and/or genomic rearrangements.

## 3. Epigenetics and Evolutionary Theory

As adaptation impacts reproductive fitness, it follows that epigenomics directly affect natural selection. It is possible to construct a chain of events: environment induces a change in the epigenome and this can lead to a modification in DNA sequence. The consequence is environmentally induced phenotypic plasticity [58]. If the change occurs in somatic cells, the resulting mutations might trigger abnormal cell behaviours and disease states. If the change occurs in germ cells, the resulting DNA mutation is passed on to subsequent generations, and this might exert a selectable phenotypic change [26]. In this context, heritable epigenetic variation could explain the faster than expected adaptation to environmental change that is often observed in natural populations [59]. Perhaps, evolutionary processes can suggest experimentally testable mechanisms by which environmental factors can influence epigenetic/genetic processes leading to a heritable change. The importance of gene control elements as drivers of evolutionary change, and particularly how they might operate during embryonic development, has been stressed [60, 61]. A recent review considers how epigenetic change might exert evolutionary effects, with emphasis on quantitative changes in gene expression due to spreading of suppressive chromatin [62].

Methylated cytosine undergoes base mutation more rapidly than unmethylated cytosine (10^−7^ per generation versus 10^8^ per generation) impacting in genomic mutagenesis [58]. It is generally accepted that there are aspects of evolutionary change that are not easily explained by the progressive accumulation of small genetic and phenotypic changes [58, 59], even if chance alone or dramatic environmental events triggering rapid changes may also play a role. Moreover, the large intraindividual epigenetic variation in the germ line may shed new light on the problem presented by one of the first geneticists, Hugo De Vries, more than a century ago, in his book *Species and Varieties: Their Origin by Mutation*, when he wrote “Natural selection may explain the survival of the fittest, but it cannot explain the arrival of the fittest.”

## 4. Conclusions and Perspectives

Stable and plastic epigenetic regulation might help researchers to understand the molecular basis of heritable and nonheritable factors, but we still need a considerable effort to get as much evidence as possible on the evolutionary bases of the epigenetic phenomenon.

During these years we highlighted the role of temperature and different ecological habitats in the evolution of genome phenotype indicating that environment can mold the genome through selection [58]. By studying animal species, which splitted millions of years ago, we had the possibility to look at epigenetic variations occurred over evolutionary times on the whole genome. Therefore, our studies represent an *in vivo *proof of the methylation-temperature-deamination hypothesis that otherwise could not be reproduced in laboratory with animal or plant models in a short time. The analysis of methylation levels in a so large number of vertebrates is the first step to understand its evolutionary meaning and functional role. Future steps should be devoted to the comparative analysis of DNA methylation in specific regions of genomes in order to understand the meaning of similarities and differences and their consequences on physiology, gene expression, and evolution.

## Figures and Tables

**Table 1:** Mammalian species analyzed for GC % and DNA methylation.

Genus	Species		GC %	5mC %	
*Tachyglossus *	*aculeatus *	∗	48.9	1	
*Ornithorhynchus *	*anatinus *	∗	48.5	1.2	Monotremes

*Monodelphis *	*domestica *	∗	39.1	0.3	
*Didelphis *	*virginiana *	∗	39.2	0.3	
*Macropus *	*rufus *	∗	41.7	0.4	
*Macropus *	*robustus *	∗	41.2	0.4	
*Vombatus *	*ursinus *	∗	40.9	0.3	
*Potorous *	*tridactyla *	∗	39.8	0.5	Marsupials

*Homo *	*sapiens *	∗	42.8	0.7	
*Tupaia *	*montana *		41.9	1.1	
*Hapalemur *	*griseus *		41.4	0.9	
*Erinaceus *	*europaeus *		45.5	0.5	
*Crocidura *	*russula *		41.4	0.7	
*Cynocephalus *	*variegatus *		40.6	0.9	
*Pteropus *	sp.	∗	40.5	1	
*Noctilio *	*albiventris *	∗	43.3	0.6	
*Artibeus *	*planirostris *	∗	47.6	0.7	
*Hipposideros *	*galeritus *		41.4	0.9	
*Rhinolophus *	*creaghi *		41.5	0.8	
*Myotis *	*lucifugus *		43.5	1	
*Chiroderma *	*salvini *		41.4	0.9	
*Nycteris *	*thebaica *		42.9	0.9	
*Chaerephon *	*pumila *		41.4	0.9	
*Manis *	sp.		42.4	0.6	
*Oryctolagus *	*cuniculus *		44.3	0.9	
*Sciurus *	*vulgaris *		39.5	0.6	
*Cricetus *	*norvegicus *	∗	40.7	0.4	
*Spalax *	sp.		38.4	0.6	
*Cavia *	*porcellus *		39.7	0.7	
*Rattus *	*norvegicus *		43.9	0.9	
*Procavia *	*capensis *		41	0.7	
*Balaenoptera *	*physalus *		41.3	0.9	
*Physeter *	*macrocephalus *		41.9	1.1	
*Phocena *	*phocena *		41.4	1	
*Canis *	*familiaris *		41.1	0.7	
*Panthera *	*uncia *		41.5	0.9	
*Equus *	*caballus *		42.8	1	
*Sus *	*scrofa *		44.6	0.8	Placentals

Values with asterisk are from Varriale and Bernardi, unpublished data.
